# Phyllodes tumor of the breast: a clinic-pathologic study of 77 cases in a Hispanic cohort

**Published:** 2015-09-30

**Authors:** Carlos Andres Ossa, Fernando Herazo, Monica Gil, Carolina Echeverri, Gonzalo Ángel, Mauricio Borrero, Jorge Madrid, Ricardo Jaramillo

**Affiliations:** 1 Grupo de Investigacion en Cancer IDC. Instituto de Cancerología, Medellin, Colombia; 2 Pathology Department. Clínica Las Américas Medellin, Colombia

**Keywords:** Breast, fibroadenoma, fibroepithelial tumor, pathology, Phyllodes tumor

## Abstract

**Introduction:**

Breast Phyllodes tumors are rare breast tumors present in less than 1% of new cases of breast cancer, usually occurring among middle-aged women (40-50 yrs).

**Objective:**

This study shows diagnostic experience, surgical management and follows up of patients with this disease during a period of ten years in a oncology referral center.

**Methods:**

Retrospectively, breast cancer registries at the institution were reviewed, identifying 77 patients with Phyllodes tumors between 2002 and 2012, who had been operated on at the Instituto de Cancerología - Clínica Las Américas, in Medellín (Colombia). Clinical and histopathological data belonging to these cases was captured and analyzed and descriptive statistics were used.

**Results:**

The follow up median was 22.5 months (IQR: 10.5-60.0), average age was 47.2 yrs (SD: 12.4), mean tumor size was 3.6 cm (SD: 4.6), 88.3% of the patients (68 cases) presented negative margins and none of them received adjuvant chemotherapy. Of the patients with Phyllodes tumors; 33.8% had benign, 31.2% had borderline and 35.0% had malignant tumor. Disease-free survival was 85.8% and overall survival was 94.5%.

**Discussion:**

Reported data in this article is in accordance with what has been reported in worldwide literature. In our cohort even the high mean size of the tumors, the risk of local relapse and metastatic disease is low than previously reported in literature. Trials with longer follow up and molecular trials in Phyllodes tumors are necessary to understand the behavior of these tumors in Hispanics population.

## Abstract (es)

**Introducción:**

Los tumores phyllodes mamarios son Tumores infrecuentes en la mama presentes en menos del 1% de los casos nuevos de cáncer mamario, por lo general ocurre en mujeres de mediana edad (40-50 años).

**Objetivo:**

Este estudio muestra la experiencia de diagnóstico, tratamiento quirúrgico y seguimiento de los pacientes con esta variedad de tumor durante un período de diez años en un centro de referencia oncológico.

**Métodos:**

Retrospectivamente, los registros de cáncer de mama en la institución fueron revisados, se identificaron 77 pacientes con tumores phyllodes entre 2002 y 2012, que habían sido operados en el Instituto de Cancerología - Clínica Las Américas, en Medellín (Colombia). Los datos clínicos e histopatológicos pertenecientes a estos casos fueron recolectados y analizados utilizando técnicas de estadística descriptivas.

**Resultados:**

La mediana de seguimiento fue de 22.5 meses (RIC: 10.5-60.0), la Media de edad fue de 47.2 años (DE: 12.4), tamaño medio del tumor fue de 3.6 cm (DE: 4.6), 88.3% de los pacientes (68 casos) presentaron márgenes negativos y ninguno de ellos recibieron quimioterapia adyuvante. De los pacientes con tumores phyllodes; 33.8% fueron benignos, 31.2% Borderline y 35.0% phyllodes maligno. Supervivencia libre de enfermedad fue 85.8% y la supervivencia global fue de 94.5%.

**Discusión:**

Los datos reportados en este artículo están acordes con lo que se ha reportado en la literatura mundial. En nuestra cohorte, a pesar de que el tamaño promedio de los tumores fue mayor, el riesgo de recidiva local y las tasas de enfermedad metastásica es menor que el reportado previamente en la literatura. Los ensayos con seguimiento más prolongado, y los ensayos moleculares en tumores phyllodes son necesarios para comprender de una manera más precisa el comportamiento de estos tumores en la población hispana.

## Introduction

The primary breast Phyllodes tumor (PT) is a rare tumor that accounts for 0.5% of all breast tumors 1. It is formed by stromal and epithelial origin tissue, which is why it is classified as part of the breast biphasic tumors 2. The original term of Phyllodes Cystosarcoma was coined by Johannes Muller in 1838 to denominate a tumor that macroscopically had the appearance of fish flesh 2. WHO in 1981 sub-classified them histologically as benign, borderline, or malignant. The incidence of benign cases of 35-64% while malignant tumors comprise 25% of the cases 2,3. Compared to the average age of patients with fibroadenoma, patients with Phyllodes tumors are diagnosed at more advanced ages, with an average of 40 to 50 yrs 1,3,4. However, the average age is lower than that of patients with ductal and lobular invasive carcinoma.

Phyllodes tumor account for 0.5% to 1.0% of all breast tumors and 2.5% of all fibroepithelial tumors. Populations-based estimates indicate that the incidence of malignant PT is 2.1 cases per million women, with the highest frequencies in Latin whites and Asians women. In the trial by Pimiento *et al*., with 124 patients with PT in the USA 43% were Hispanics with higher percentage of borderline and malignant tumors in Hispanics patients (*p* <0.001). In this trial Hispanics patients tended to have large tumors and higher mitotic rates.

In Colombia does not exist publications with the characteristic of the PT in our patients, for what in this article describes clinical and pathologic characteristics and treatment of patients with PT in a Colombian comprehensive cancer center. 

## Materials and Methods

Cases were captured from the Breast Cancer Institutional Registry of the Instituto de Cancerología (IDC)-Clínica Las Américas in Medellín, during the period between 2002 and 2012 when 81 patients with PT histologic diagnosis were treated. Inclusion criteria were patients over 18 yrs, pathologic report of PT, treated at the IDC. Patients with final fibroadenoma diagnosis, PT that had associated *in situ* or infiltrating carcinoma, and patients younger than 18 were excluded.

The clinical histories of those patients were reviewed. Demographic variables, such as age, size, and histological level were recorded in a database. Clinical and pathological characteristics, treatments undertaken, and recurrence date were also recorded.

**Figure 1.**
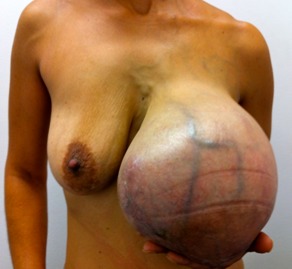
Patient with 35 yrs old, malignant Phyllodes Tumor initial presentation. Weight: 3,250 g. Receive radical surgery and radiotherapy. Dead after 32 months of metastatic disease.

**Figure 2.**
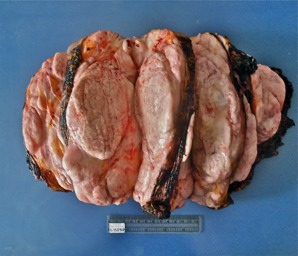
Macroscopic analysis malignant Phyllodes Tumor corresponding to the case described in the previous photo. Measure 85 x 58 mm on cut section have a red or grey "meaty" consistency with fibrogelatinous, hemorrhagic, and necrotic areas with leaf like protrusions into the cystic spaces.

**Figura 3.**
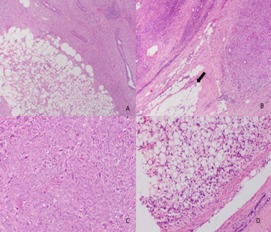
Malignant Phyllodes Tumor. **A.** H&E staining. x25 Low-zoom micrograph that shows the fibroepitelial component of the injury. **B.** x4 infiltrating edge, given by the presence of tumor focus further than the tumor capsule infiltrating the fat(. **C.** x20 high cellularity of the stromal component with the presence of mitosis. **D.** x20 presence of liposarcoma as heterologous component .

All the histologic slides were re-examined by any of our breast pathologist in the Pathology Service of the institution and were classified as benign, borderline, or malignant, in compliance with the criteria proposed by Azzopardi and Salvadori and adopted by WHO 2. These criteria are: tumor margin (defined or infiltrating), stromal cellularity (slight or severe), stromal overgrowth (absent, slight, severe), tumor necrosis (present or absent), cellular atypia (absent, slight, severe), and number of mitotic fields per ten high-power fields 2 (Figs. 1, 2  and 3 ).

Data was analyzed using Stata^®^ V12 software, performing main trend measure analysis, bivariate analysis, and survival curves according to the histological level.

## Results

During the period of the study, at the IDC a total of 81 patients with PT diagnosis were present, out of which 77 cases were included corresponding to 95.1% of the total. Four cases were excluded because they did not comply with inclusion criteria, three of them because they presented ductal components and the other one because she was younger than 18 yrs.

Ninety seven point four percent (n= 75) of the patients lived in Antioquia department. The mean age of patients was 47.2 yrs (standard deviation, SD: 12.4). According to the histological analysis, 35% (n= 27) of the cases were found to be benign PT, 31.2% (n= 24) borderline PT, and 33.8% (n= 26) malignant PT. Tumors were 60.5% (n= 46) measured over 5 cm at the moment of the diagnosis; the mean for the benign PT was 19.4 mm (SD: 32.4, range= 1-120 mm), 39.1 mm for the borderline (SD: 35.2, range= 2.5-180.0), and 45.5 mm for the malignant (SD: 48.6, range= 2-180). As far as the number of mitosis, 27 cases (36.5%) presented 0-4 mitosis, 25 cases (33.8%) presented 5-10 mitosis, and 22 cases (29.8%) presented more than 10 mitosis. Only three of the cases studied (3.9%), presented heterologous elements at the moment of the definite pathological evaluation. 

Every case was managed surgically; 50.7% (n= 39) were managed with conservative surgery and the rest with radical surgery and 9.1% of the cases (n= 7), used axillar dissection procedures because an infiltrating component or an epithelial histology tumor was suspected. In every case, the axillary emptying was negative.

Of the 38 patients undergoing mastectomy, 52.6% had mammary reconstruction (immediate or deferred); 13% of the cases had some sort of complication, being the most frequent flap necrosis (7.8%), bleeding in two cases, and surgery site infection in two cases (2.6%) (Table 1).

**Table 1.** Characteristic of Phyllodes tumors in Instituto de Cancerología.

Characteristic	Benign*	Borderline*	Malignant*	*p *value
Sample number	26	24	27	
Mean age (yrs)	43	49	50	0.071
Laterality				
Right	13 (50)	7 (29)	12 (44)	
Left	13 (50)	17 (71)	15 (56)	0.305
Surgical management				
Mastectomy	4 (15)	10 (42)	24 (89)	
Conservative surgery	22 (85)	14 (58)	3 (11)	
Tumor characteristics				
Mean size (cm)	2.1	4.2	4.5	
± sd	3.3	5.1	5.0	0.132
Mitoses				
0-4	22 (84)	4 (17)	1 (4)	
5-10	2 (8)	17 (71)	6 (22)	
>10	1 (4)	1 (4)	20 (74)	
Not available	1 (4)	2 (8)		
Margins				
Positive	2 (8)	1 (4)	5 (8)	
Negative	24 (92)	22 (92)	22 (92)	
Not available	-	1 (4)	-	0.265
Radiotherapy				
Yes	-	6 (25)	17 (63)	
No	-	18 (75)	10 (37)	

*n(%)

Twenty-nine point nine percent 29.9% (n= 23) of the patients received radiotherapy. Out of these cases, six patients had borderline PT (n= 6%) and 16 patients had malignant PT (73.9%). None of the patients received systemic chemotherapy in an adjuvant manner. Seven point eight percent 7.8% of the patients (n= 6), presented disease progression, two of them with local relapse and the four remaining cases with systemic relapse, the lung being the most frequent relapse (3 cases). A patient suffered a tumor progression to the central nervous system. No progression to the liver was documented in any of the cases. Two patients (2.5%) received systemic chemotherapy to manage their metastatic progression with schemes based on Doxorubicin and Dacarbacin.

A patient with initial histologic diagnosis of benign PT, with a tumor of 8 cm (diameter), presented two local relapses, both of them as borderline PT. She was managed with local re-excision for the first relapse, and then mastectomy with reconstruction for the second. She then received radiotherapy. Out of the six patients (16.7%) presented loco-regional relapse. One case was initially managed with conservative surgery and five patients with radical surgery. 

Out of the 20 patients who underwent mammary reconstruction, two of them presented tumor relapse and the remaining 18 were healthy at the moment of the last control.

The follow up median was 22.5 months (IQR= 10.5-60.0). The disease-free survival (DFS) rate for the complete group of patients was 85.8% [95% CI: 70.9-93.4] and the overall survival (OS) rate was 94.5% (95% CI: 73.7-95.0). The follow up loss was 7.8% (6 cases).

**Figura 4.**
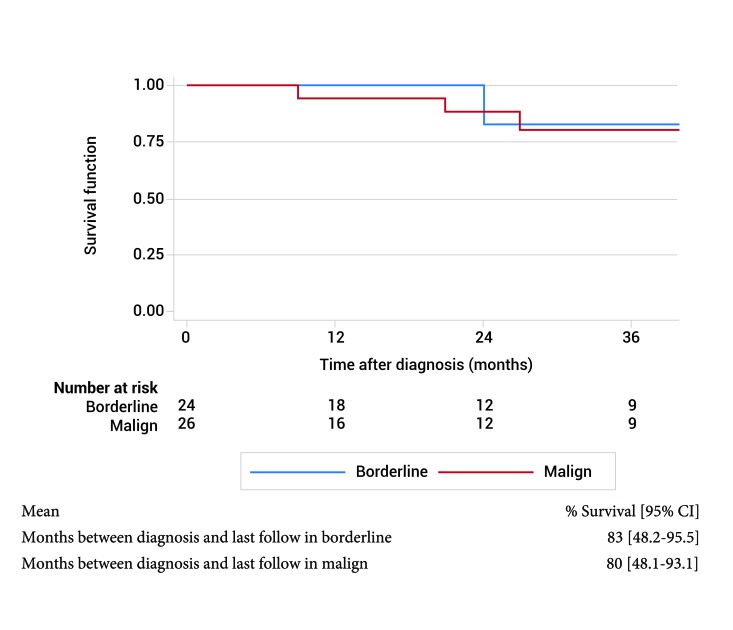
Overall survival histological type.

**Figure 5.**
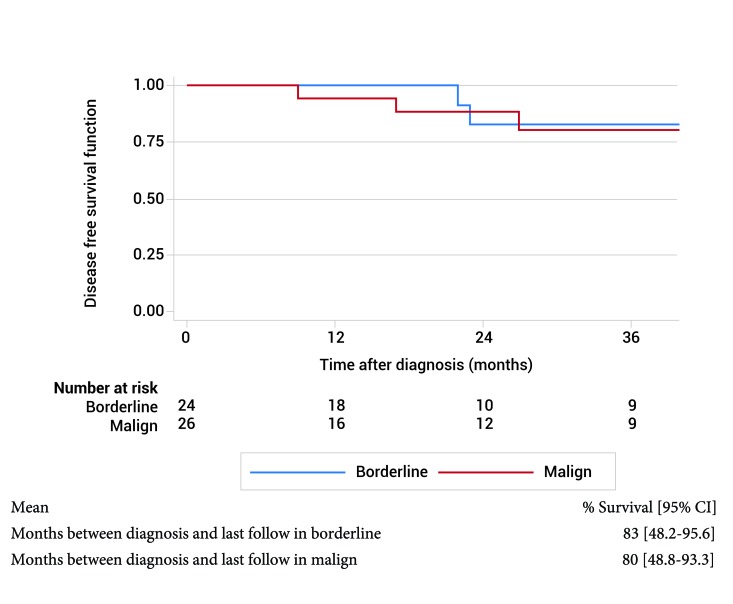
Disease free survival by histological type.

In the overall survival the months between diagnosis and last follow in borderline is 83% (95% CI: 48.2-95.5) and malignant tumor is 80% (95% CI: 48.1-93.1). In disease free survival the months between diagnosis and last follow in borderline is 83% (95% CI: 48.2-95.6) and malign tumor is 80% (95% CI: 48.8-93.3). OS and DFS by borderline and malignant type PT are shown in the Figures 4 and 5. 

## Discussion

The current study with 77 cases, is one of the largest breast mammary PT series reported in Latin America 5-8, with a global survival of 94.5% that is similar to that reported in other series found in the world literature 9; this survival data was not affected despite the large tumor size of our patients at the moment they received oncological management (60.5% of the patients had tumors larger than five centimeters); however, the radical surgery rate in our cohorts were greater than those reported in other series 10-12)

Given the infrequency of this type of tumor, epidemiological data is scarce. Berstein *et al*., 13 in the city of Los Angeles over a 17-year period, the average annual incidence was 2.1 cases per million women; this study reported a high incidence in Latin white women compared to non-Latin white, Asian, and black.

Breast PT have a diverse range of biological behaviors, from variants benign behavior to variants with the capacity to generate distant metastasis, including the capacity to histologically de-differentiate them in sarcomatose injuries with absence of epithelial component 10,14. Given the lack of nomenclature uniformity, the WHO recommended in 1982 that every injury must be denominated as PT, which has been widely accepted 2, 15.

The recommended treatment for these tumors is surgical resection with tumor-free margins of one centimeter or larger. As long as this condition is complied with, the breast conservative surgery is the preferred surgical treatment. Simple mastectomy is recommended if the negative margins cannot be guaranteed through conservative surgery. In our series, 88.1 % (n= 67) of the tumors measured over three centimeters, so 49.3% (n= 38) of the cases underwent mastectomy. In Surveillance (SEER) database in the United States of America, just 8 out of 498 PT cases with known ganglionic stage presented axillar compromise 1. In our series, none of the cases was documented as compromising or with axillar progression. Adjuvant therapy with radiotherapy, chemotherapy, or both, has not played a clear and definite role in the treatment of PT, existing contradictory results in the literature 11,16,17. In our institution, as a group policy, systemic management is not offered to PT and management with radiotherapy is recommended for patients with malignant PT or with borderline injuries over five centimeters.

The most effective surgery for PT is wide local resection with a histological margin larger than one centimeter, which is much larger than the one recommended for breast infiltrating ductal injuries and *in situ* 3. Unfortunately, local resection without attention to the margins is frequently performed, particularly due to the fact that these cases are pre-surgery sub-diagnosed as fibroadenomas 12,14; some of them reach large sizes (Fig. 1) that are worth mastectomies to achieve adequate oncological control which was necessary in 49.3% of our patients, a proportion we consider high compared to other series like Spitalieri et al in 2013 that was 20% 9.

Relapse rates are unacceptably high after local resection or enucleation of the injury without adequate margin. Several reports indicate that wide local resection generates local relapse rates of 8% for benign PT and 21-36% for borderline and malignant tumors 9,10). In our caseload, local relapses were 7.8% which is under published series. In a retrospective case series of 48 women with malignant PT and nine-year average follow up, recurrence rate was 60% for those treated with local resection compared to 28% for those treated with local resection and appropriate margins (over one centimeter). Local relapse and specific survival associated to cancer, was related to the tumor size and the resection margins 18.

There is no published evidence in the literature that assesses the risk of immediate mammary reconstruction in patients with PT in terms of local relapse and survival. In our series, 20 cases underwent reconstruction; 11 cases with wide dorsal muscular flap with prosthesis, five cases with abdominal straight muscular flap reconstruction (Tram), and four cases with pectoral muscular flap. It was not possible to demonstrate that patients with mammary reconstruction had a worse oncologic forecast than non-reconstructed in terms of global survival and disease-free.

In respect of the use of radiotherapy on PT, a large retrospective series of patients with malignant PT treated just with surgical resection revealed sub-optimal five-year control rates (79% in 169 patients treated with local resection vs. 91% in 207 patients treated with mastectomy) 19. In this series, the authors concluded that patients with malignant PT over two centimeters in diameter treated with just local resection, adjuvant radiotherapy must be strongly recommended as the treatment option. An important limitation of the study is the lack of information about the condition of the margins. 

In another European series of 443 women published by Balkacemi *et al*., 11, patients treated for PT, radiotherapy was associated with ten-year local control rates between 59% (without radiotherapy) and 86% (with radiotherapy) for borderline and malignant PT.

Taken together, these results indicate that radiotherapy seems to be effective in reducing recurrence rates after a conservative surgery for borderline and malignant PT. Some groups argue that adjuvant radiotherapy is appropriate when it is not possible to obtain a margin larger than or equal to one centimeter. 

Our center does not have a uniform policy to use radiotherapy on every borderline and malignant PT; however, according to the results of the current study, the proposal is to offer it to borderline PT with conservative surgery and/or tumors larger than five centimeters and on malignant PT.

It is important to mention that some tumors, due to their large size, despite being managed with mastectomy, are not able to achieve over one-centimeter surgical margin so radiotherapy must be adequate as well for these cases 11. The case of the benign PT that received radiotherapy, relates to a case that presented two tumor relapses and it was proposed by the medical board to consider the tumor as borderline PT behavior, offering, then, radiotherapy management. 

The benefit of adjuvant chemotherapy is controversial. There are no prospective or randomized studies of adjuvant chemotherapy on this type of tumor. A Mexican observational study published by Morales-Vásquez 16 included 28 patients with malignant PT treated in a period of ten years (1993-2003) with Doxorubicin and Dacarbacin compared to observation. After the surgical resection, the treatment was based on the patient's election. There was no local relapse or global survival difference in these two groups of patients. However, this is a small, retrospective, uncontrolled study that did not use Ifosfamide, which is a medication superior to Dacarbacin that is currently the standard to manage other sarcomas. As of today, international management guidelines do not recommend the use of routine chemotherapy for this group of patients and must be considered for patients with high relapse risk (high level or large size tumors and young patients) 20. Our oncological center considers systemic chemotherapy as poor benefit as adjuvant and it is reserved to manage metastatic disease. 

## Conclusion

We believe that the good results achieved in our study in OS and DFS are due to the high rate of radical surgery vs conservative surgery in the group of patients with malignant PT (89% vs 11%) leaving the radiotherapy only in cases where surgical margins may not be adequate (greater than one centimeter) in our experience breast reconstruction is not a contraindication for this group of tumors.

We could not identify prognostic differences for borderline and malignant tumors as classified by the WHO; therefore is urgently needed develop some molecular studies in this group of tumors, that allow us to better understand the biological behavior and the ability to perform metastasis in PT.
